# Diffuse Alveolar Hemorrhage as a Life-Threatening Complication of Systemic Lupus Erythematosus: A Case Report

**DOI:** 10.7759/cureus.73586

**Published:** 2024-11-13

**Authors:** Fatema Jamsheer, Sharifa Alzayani, Njood Alsudairy

**Affiliations:** 1 College of Medicine, Royal College of Surgeons in Ireland, Busaiteen, BHR; 2 College of Medicine, Arabian Gulf University, Manama, BHR; 3 Radiology, The Second Health Cluster, Jeddah, SAU

**Keywords:** autoimmune disease, case report, cyclophosphamide, diffuse alveolar hemorrhage, hemoptysis, high-dose corticosteroids, immunosuppressive therapy, intensive care, respiratory failure, systemic lupus erythematosus

## Abstract

Diffuse alveolar hemorrhage (DAH) is a rare but severe pulmonary complication in systemic lupus erythematosus (SLE), characterized by alveolar bleeding leading to respiratory distress, hypoxemia, and often hemoptysis. Rapid diagnosis and aggressive immunosuppressive therapy are crucial for survival. A 55-year-old woman with a five-year history of SLE presented with acute dyspnea, hemoptysis, pleuritic chest pain, fatigue, and low-grade fever. On examination, she had bilateral crackles on auscultation, hypoxemia, tachycardia, and mild pitting edema. Laboratory tests revealed anemia, elevated anti-double-stranded DNA (anti-dsDNA) titers, and low complement levels. Imaging studies showed bilateral patchy infiltrates on chest X-ray and ground-glass opacities on high-resolution CT (HRCT), suggesting DAH. Differential diagnoses considered included lupus pneumonitis, infection, and pulmonary embolism, but DAH was strongly suspected based on the patient’s clinical presentation, lab, and imaging findings. Treatment with high-dose intravenous methylprednisolone and cyclophosphamide was initiated, leading to stabilization of respiratory symptoms and gradual improvement. The patient was discharged on oral corticosteroids and hydroxychloroquine, with significant clinical improvement observed at one-month follow-up. DAH in SLE is a medical emergency that requires a multidisciplinary approach and rapid therapeutic intervention. This case underscores the potential for favorable outcomes when DAH is recognized and managed promptly, though further research is needed to refine long-term treatment strategies for these high-risk patients.

## Introduction

Diffuse alveolar hemorrhage (DAH) is a rare but life-threatening pulmonary complication that can occur in systemic lupus erythematosus (SLE), an autoimmune disease affecting multiple organ systems. Characterized by acute bleeding into the alveolar spaces, DAH often presents with hemoptysis, anemia, hypoxemia, and diffuse pulmonary infiltrates on imaging [1,2]. Although hemoptysis is a hallmark symptom, it may be absent in up to one-third of cases, complicating diagnosis. The underlying mechanism of DAH in SLE is thought to involve immune complex-mediated damage to the pulmonary microvasculature, resulting in increased permeability and alveolar bleeding [2,3].

DAH in SLE is associated with high morbidity and mortality, especially without prompt recognition and treatment. Managing DAH in lupus is challenging and requires a multidisciplinary approach, including supportive care and aggressive immunosuppression. High-dose corticosteroids are typically first-line therapy, often combined with cyclophosphamide or other immunosuppressive agents to control the underlying inflammation rapidly. Despite treatment, recurrence is common, and long-term outcomes are variable [1-3].

This report presents the case of a 55-year-old woman with SLE who developed DAH, detailing her clinical course, diagnostic work-up, and management. This case emphasizes the importance of early identification and intervention in DAH secondary to SLE, highlighting clinical considerations for improving patient outcomes in this complex condition.

## Case presentation

The patient, a 55-year-old woman, presented to the emergency department with acute onset dyspnea, hemoptysis, and pleuritic chest pain. She reported a two-week history of worsening shortness of breath, initially attributed to an upper respiratory tract infection. However, in the days preceding her admission, her symptoms progressively worsened, with increased frequency and quantity of hemoptysis, accompanied by fatigue, anorexia, and low-grade fever. The patient denied recent travel, exposure to sick contacts, or occupational hazards. She was a non-smoker with no history of illicit drug use, and her only medication was hydroxychloroquine, taken regularly for her known diagnosis of systemic lupus erythematosus (SLE), diagnosed five years prior. Her SLE had been stable with no history of lupus nephritis, thrombotic events, or significant respiratory symptoms.

On examination, the patient appeared pale, fatigued, and in mild respiratory distress. Vital signs included a heart rate of 115 beats per minute, respiratory rate of 24 breaths per minute, blood pressure of 98/60 mmHg, and an oxygen saturation of 88% on room air. Auscultation of her lungs revealed diffuse bilateral crackles with no wheezing, and there was slightly decreased air entry in the lower lung zones. Cardiovascular examination showed tachycardia with a regular rhythm but no murmurs. Abdominal examination was unremarkable, with no palpable hepatosplenomegaly. On skin examination, a malar rash was noted across her cheeks, and mild pitting edema was observed in the lower extremities.

Laboratory investigations revealed a hemoglobin level of 7.8 g/dL, down from a baseline of 11.2 g/dL. Platelet count was mildly decreased at 120 x 10^3/µL, and white blood cell count was within normal limits. Renal function tests showed a serum creatinine level of 1.4 mg/dL, mildly elevated from her baseline of 0.8 mg/dL. Liver enzymes were within normal range. Arterial blood gas analysis demonstrated hypoxemia with a partial pressure of oxygen (PaO2) of 62 mmHg on room air. The coagulation profile was normal, with an international normalized ratio (INR) of 1.1 and activated partial thromboplastin time (aPTT) within the reference range. Urinalysis showed microscopic hematuria but no proteinuria or red cell casts. Autoimmune panel revealed positive anti-double-stranded DNA (anti-dsDNA) antibodies at a high titer, low complement levels (C3 and C4), and elevated anti-Smith antibodies, consistent with active SLE. Serum ANCA (anti-neutrophil cytoplasmic antibodies) was negative.

**Figure 1:**
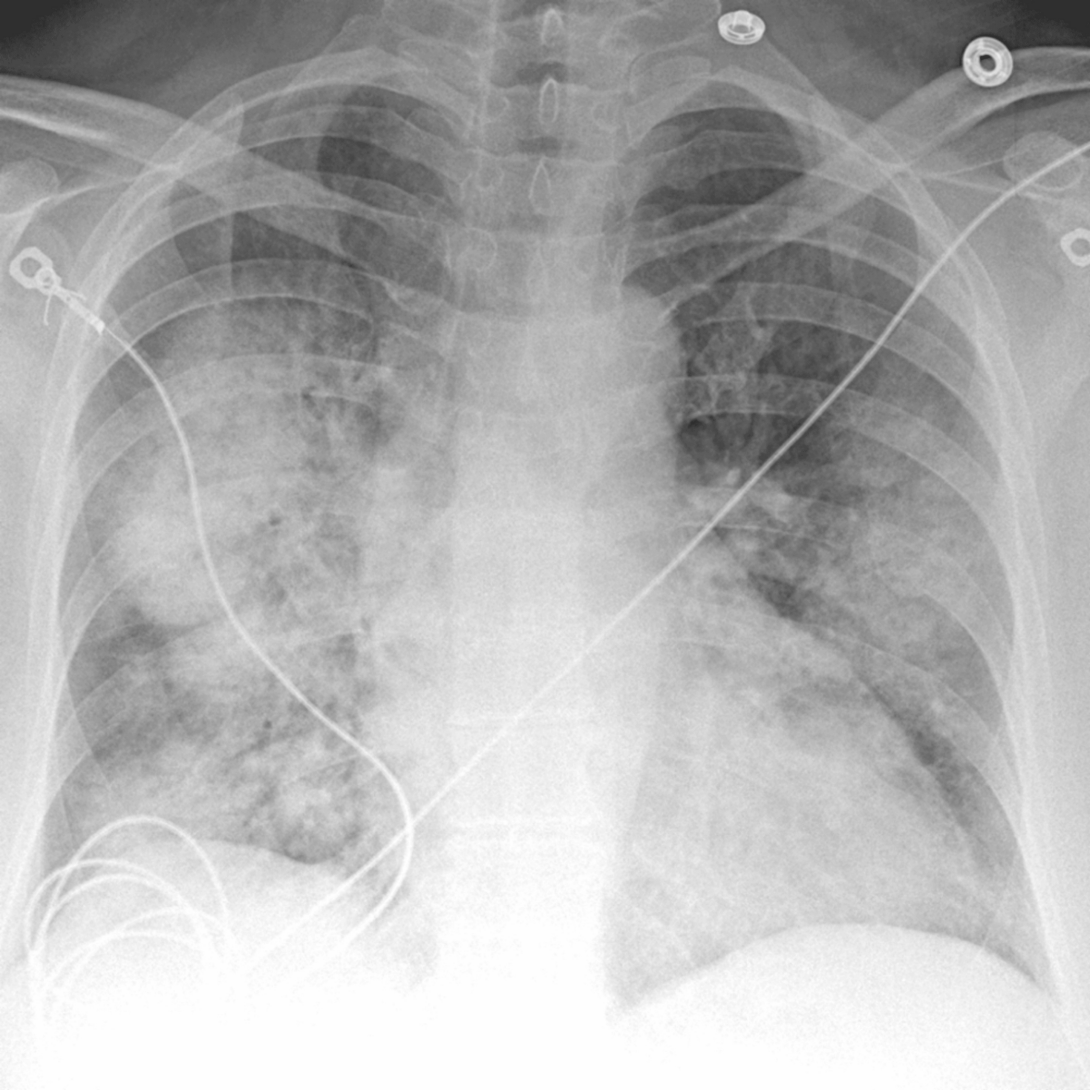
Frontal chest radiograph showing asymmetric, diffuse, bilateral heterogeneous airspace disease and consolidations.

**Figure 2:**
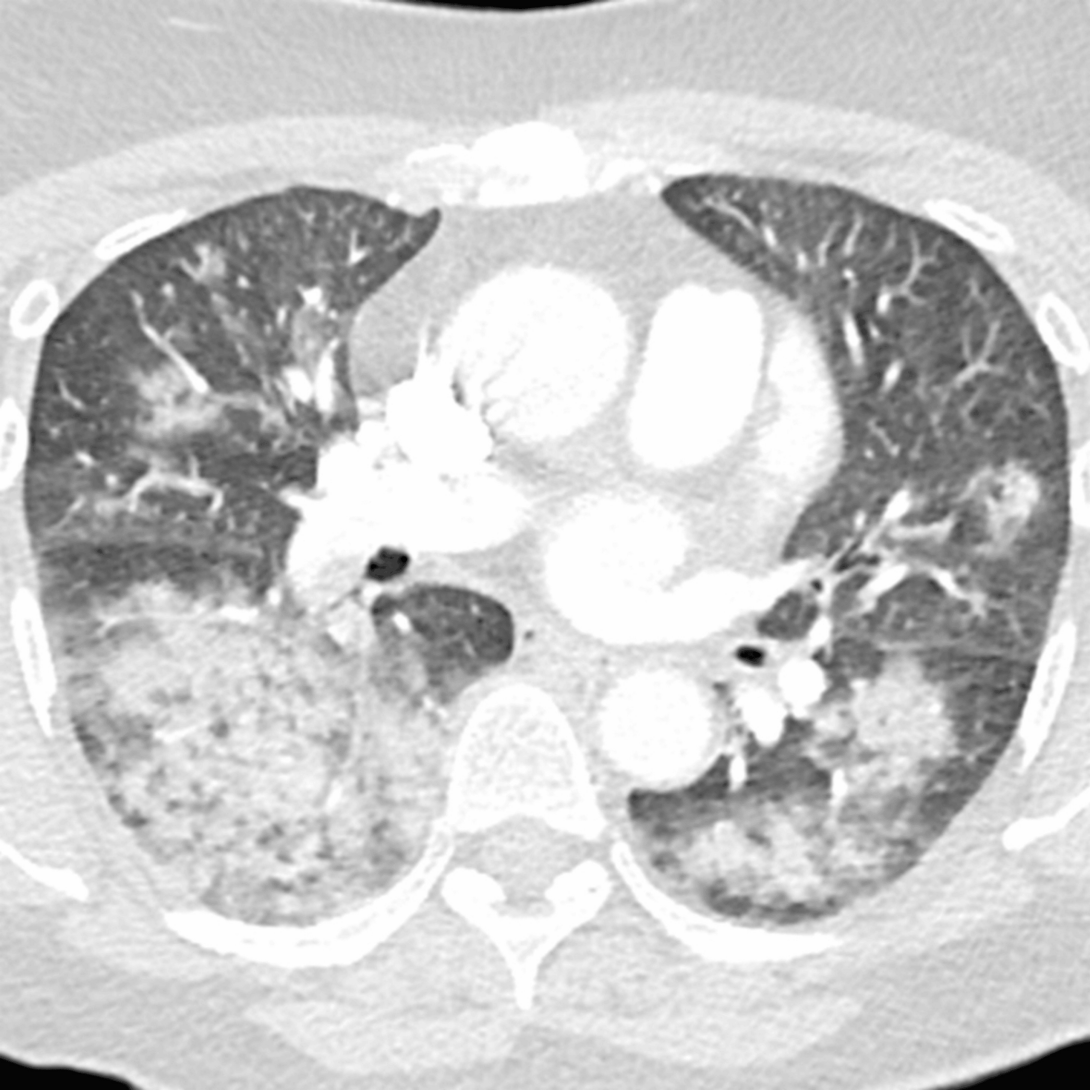
Axial CT showing multifocal airspace disease and ground-glass opacities bilaterally. The imaging findings are consistent with diffuse alveolar hemorrhage.

A chest X-ray revealed bilateral patchy infiltrates, predominantly in the perihilar regions, raising suspicion for diffuse alveolar hemorrhage (Figure 1). High-resolution computed tomography (HRCT) of the chest demonstrated extensive ground-glass opacities and consolidations bilaterally, consistent with alveolar filling processes (Figure 2). Given her known history of SLE, acute presentation, and imaging findings, diffuse alveolar hemorrhage (DAH) secondary to lupus was strongly suspected. Differential diagnoses included acute lupus pneumonitis, infection, and pulmonary embolism; however, DAH was favored due to her clinical presentation, rapid drop in hemoglobin, and imaging findings. Pulmonary embolism was excluded via CT pulmonary angiogram, which showed no evidence of thromboembolic disease.

The patient was admitted to the intensive care unit for close monitoring and supportive care. She received supplemental oxygen, initially at 5 liters per minute via nasal cannula, which was escalated to high-flow oxygen due to worsening hypoxemia. Immunosuppressive therapy was initiated promptly, given the high suspicion for DAH secondary to SLE. High-dose intravenous methylprednisolone (1 g daily for three days) was administered as pulse therapy, followed by a gradual taper. Additionally, intravenous cyclophosphamide was given to achieve rapid immunosuppression, aiming to control the underlying inflammatory process.

The hospital course was notable for persistent hemoptysis and anemia, necessitating transfusions of packed red blood cells. After three days, her respiratory status improved, and oxygen requirements decreased. The patient’s hemoglobin stabilized, and repeat imaging demonstrated partial resolution of the ground-glass opacities. Over the next week, she continued to show clinical improvement, and a plan for transition to oral prednisone was made.

The patient was discharged on day 12 with oral prednisone and hydroxychloroquine, with instructions to follow up with her rheumatologist within one week. At the one-month follow-up, she reported no further hemoptysis or dyspnea, and a repeat chest X-ray showed complete resolution of previous infiltrates. Laboratory tests revealed normalized complement levels, improved hemoglobin, and stable renal function.

## Discussion

This case highlights DAH as a rare but severe pulmonary manifestation of SLE, emphasizing the need for prompt recognition and aggressive treatment. DAH is reported to occur in approximately 1-5% of SLE patients, often presenting with life-threatening respiratory distress, hemoptysis, and hypoxemia, as seen in this 55-year-old patient. The clinical picture can vary, with some patients lacking hemoptysis, as was initially seen in this case, which may delay diagnosis [2-4]. In our patient, early imaging and laboratory findings of acute anemia, ground-glass opacities on chest CT, and high-titer anti-dsDNA antibodies were instrumental in raising suspicion of DAH. Studies underscore the importance of rapid diagnostic work-up, as delays in diagnosis and treatment are associated with significantly higher mortality rates.

From a pathophysiological standpoint, DAH in SLE is thought to be related to immune complex deposition within the alveolar capillaries, leading to complement activation and inflammatory damage to the alveolar walls. This immune-mediated vascular injury results in the permeability of the alveolar-capillary membrane, leading to hemorrhage. Low complement levels, high-titer anti-dsDNA antibodies, and active SLE markers in our patient’s case were consistent with a heightened autoimmune response, which likely precipitated the DAH episode [1,3]. This is consistent with findings from studies showing that active disease status, marked by increased anti-dsDNA titers and decreased complement, correlates with an increased risk of DAH. Therefore, close monitoring of these markers in SLE patients may help identify those at higher risk [4-6].

In terms of management, the patient’s treatment aligns with evidence-based approaches, utilizing high-dose corticosteroids and cyclophosphamide to control inflammation. Pulse methylprednisolone is commonly used to suppress the immune response acutely, while cyclophosphamide or other immunosuppressants, such as mycophenolate mofetil, are typically added for their steroid-sparing effects and efficacy in controlling severe lupus manifestations. Studies support this combination as the standard for managing DAH in lupus, especially in patients with extensive alveolar hemorrhage. Rituximab has also been explored as an adjunctive therapy for refractory DAH in SLE, with some promising results [1-4]. In this case, our patient’s positive response to methylprednisolone and cyclophosphamide underscores the efficacy of these agents in acute control; however, further research is warranted to optimize long-term management strategies and assess the role of newer therapies in this patient population.

The patient’s hospital course, marked by initial stabilization followed by a gradual taper of corticosteroids, highlights the complexities of managing DAH in SLE. Although her condition improved with this approach, recurrence is a well-documented concern, with studies reporting relapse rates as high as 50%. These findings emphasize the need for close follow-up and possible long-term immunosuppressive therapy to prevent recurrence. Additionally, the prognosis for SLE-related DAH remains guarded, with mortality rates ranging between 20-50%, underscoring the need for early recognition and aggressive intervention.

This case contributes to the broader understanding of DAH in SLE by reinforcing key diagnostic and therapeutic challenges and emphasizing the importance of individualized care. It serves as a reminder of the variability in clinical presentation and the potential for rapid clinical deterioration, which warrants vigilance in high-risk patients. Furthermore, the successful outcome in this patient, achieved through a prompt diagnosis and a multidisciplinary approach, aligns with recommendations in the literature for managing complex SLE manifestations and highlights the potential for positive outcomes with timely intervention.

## Conclusions

In conclusion, this case underscores the critical importance of early recognition and aggressive management of DAH in patients with SLE. DAH is a rare but severe complication associated with high morbidity and mortality, necessitating prompt intervention to prevent rapid deterioration. This case highlights the effectiveness of high-dose corticosteroids and cyclophosphamide in controlling the acute inflammatory process, as well as the need for vigilant follow-up to monitor for recurrence and long-term complications. Ultimately, a multidisciplinary approach, incorporating timely diagnosis, immunosuppressive therapy, and close monitoring, is essential for optimizing outcomes in SLE patients with DAH. Further research into additional therapeutic options and long-term management strategies could provide deeper insights into improving the prognosis for this challenging condition.
